# Genome Engineering in Plant Using an Efficient CRISPR-xCas9 Toolset With an Expanded PAM Compatibility

**DOI:** 10.3389/fgeed.2020.618385

**Published:** 2020-12-16

**Authors:** Chengwei Zhang, Guiting Kang, Xinxiang Liu, Si Zhao, Shuang Yuan, Lu Li, Yongxing Yang, Feipeng Wang, Xiang Zhang, Jinxiao Yang

**Affiliations:** Beijing Key Laboratory of Maize DNA Fingerprinting and Molecular Breeding, Beijing Academy of Agriculture & Forestry Sciences, Beijing, China

**Keywords:** genome editing, xCas9, cytosine base editor, tRNA-sgRNA, rice

## Abstract

The CRISPR-Cas9 system enables simple, rapid, and effective genome editing in many species. Nevertheless, the requirement of an NGG protospacer adjacent motif (PAM) for the widely used canonical *Streptococcus pyogenes* Cas9 (SpCas9) limits the potential target sites. The xCas9, an engineered SpCas9 variant, was developed to broaden the PAM compatibility to NG, GAA, and GAT PAMs in human cells. However, no knockout rice plants were generated for GAA PAM sites, and only one edited target with a GAT PAM was reported. In this study, we used tRNA and enhanced sgRNA (esgRNA) to develop an efficient CRISPR-xCas9 genome editing system able to mutate genes at NG, GAA, GAT, and even GAG PAM sites in rice. We also developed the corresponding xCas9-based cytosine base editor (CBE) that can edit the NG and GA PAM sites. These new editing tools will be useful for future rice research or breeding, and may also be applicable for other related plant species.

## Introduction

The clustered regularly interspaced short palindromic repeats (CRISPR)/CRISPR-associated nuclease 9 (Cas9) system derived from microbial adaptive immune systems has facilitated diverse genomic manipulations, including targeted gene disruption (Bortesi and Fischer, 2015; Ma et al., 2015; Xie et al., 2015), transcriptional activation or repression (Lowder et al., 2015; Piatek et al., 2015), and base substitutions (Li et al., 2017; Lu and Zhu, 2017; Zong et al., 2017), in various organisms and cell types (Komor et al., 2017; Ge et al., 2019). The application of these genomic modifications has led to substantial advances in research regarding plant biology as well as crop breeding (Yin et al., 2017; Hille et al., 2018). However, to be recognized by Cas9, a target site requires a short protospacer adjacent motif (PAM) sequence at its 3′ end (Mojica et al., 2009). The widely used Cas9 from *Streptococcus pyogenes* (SpCas9) mainly recognizes an NGG PAM sequence (Sternberg et al., 2014), thereby restricting the targetable sites in the genome.

To address this limitation, researchers have used natural CRISPR nucleases with different PAM requirements, including those of *Neisseria meningitides* (NmeCas9) (Esvelt et al., 2013), *Streptococcus thermophilus* (StCas9) (Xu et al., 2015), *Staphylococcus aureus* (SaCas9) (*Ran et al.*, *2015*), *Campylobacter jejuni* (CjeCas9) (Kim et al., 2017), and *Geobacillus thermodenitrificans* (ThermoCas9 and GeoCas9) (Harrington et al., 2017; Mougiakos et al., 2017). However, the PAMs recognized by these Cas9s are relatively complex, restricting the widespread use of these nucleases for genome editing. To date, only SaCas9 has been commonly applied in plants (Kaya et al., 2016; Qin et al., 2019). In addition to the Cas9 proteins, class 2 type V CRISPR-Cas systems involving Cas12a (or Cpf1) and Cas12b (or C2c1) have been adopted for modifying genomes at AT-rich PAM sequences (Zetsche et al., 2015; Teng et al., 2018). For example, LbCpf1 and FnCpf1 can modify the genomes of many plant species (Endo et al., 2016; Tang et al., 2017).

Another way to address the limitations of the CRISPR-Cas9 system related to the PAM sequence involves altering the PAM-interacting domain of Cas9. Several engineered SpCas9 and SaCas9 variants that can recognize NGA (VQR), NGCG (VRER), NGAG (EQR), and NNNRRT (SaKKH-Cas9) PAM sequences have been obtained (Kleinstiver et al., 2015a, Kleinstiver et al., 2015b). Nevertheless, the PAMs recognized by these Cas9 proteins are still relatively complex. Some engineered Cas9 variants with an increased PAM compatibility have recently been reported, including the SpCas9 variants, xCas9 and Cas9-NG, which enable researchers to target simple non-canonical NG PAM sites; these variants have robust editing activities without sacrificing DNA specificity in human cells (Hu et al., 2018; Nishimasu et al., 2018). Moreover, xCas9 is functional over a relatively broad range of PAM sequences, including GAA and GAT, which are not recognized by Cas9-NG. At least six groups have employed xCas9 to manipulate the rice genome (Hua et al., 2019; Li et al., 2019; Ren et al., 2019; Wang et al., 2019a; Zhong et al., 2019; Zeng et al., 2020). However, the xCas9-based gene disruption system is unexpectedly inefficient for targeting sites with GAA and GAT PAM sequences in transgenic T_0_ plants. In the current study, we developed an efficient CRISPR-xCas9 system in rice that can recognize GAA, GAT, and even GAG PAM sites without sacrificing NG PAM recognition. We also developed the corresponding xCas9-based cytosine base editor (CBE), which enables the efficient C-to-T conversion at GA and NG PAM sites in rice.

## Materials and Methods

### Plasmid Construction

In this study, the codons encoding the SpCas9 protein described by Cong et al. (2013) were first optimized for rice by GenScript Corp. (Nanjing, China). The following mutations were introduced by PCR to obtain xCas9: A262T/R324L/S409I/E480K/E543D/M694I/E1219V (Supplementary Data). The SpCas9n & PmCDA1 & UGI & T35s sequence (Wu et al., 2019) was replaced by the xCas9 & T35s fusion sequence between the SnabI and AvrII restriction enzyme sites in the SpCas9n-pBE-basic vector to generate pxCas9-basic-M. Next, pxCas9-basic-M was digested with BsaI and HindIII, after which the larger fragment lacking the sgRNA & *OsU3* terminator was purified and ligated to the esgRNA & poly-T fragment, which was digested with BbsI and HindIII to generate pxCas9-basic. Using the method described by Ma et al. (2015), targets were added to pxCas9-basic to generate pxCas9 constructs. Each pxCas9 construct comprised three target sequences, respectively, under the control of the *OsU3, OsU6c*, and *OsU6a* promoters. The pxCas9 constructs were digested with SnabI and AvrII, after which the xCas9 & T35s fragment was replaced by xCas9n & PmCDA1 & UGI & T35s and ecTadA & ecTadA^*^ & xCas9n & T35s sequences, ultimately generating the xCas9n-CBE and xCas9n-ABE (adenine base editor) constructs, respectively. Regarding the vector for pxCas9 without a tRNA, pxCas9-basic-M was digested with BamHI and HindIII, after which the larger fragment lacking the tRNA & sgRNA & OsU3 terminator was purified and ligated to the esgRNA & poly-T fragment, which was digested with BamHI and HindIII to generate pxCas9-no tRNA-basic. Three target sequences were then added using primers lacking a tRNA sequence as described by Ma et al. (2015). All of the primers used in this study are listed in Supplementary Table 1.

### Rice Transformation

All of the constructed binary vectors were inserted into *Agrobacterium tumefaciens* strain EHA105 cells using a freeze/thaw method. The transformed *A. tumefaciens* cells were then used to infect rice embryogenic calli induced from mature Nipponbare rice seeds as previously described (Hiei and Komari, 2008). After 10 min, the calli were recovered for 3 days and then cultured on selection medium containing 50 μg/mL hygromycin for 4 weeks to obtain hygromycin-resistant calli. The transgenic calli were then cultured on regeneration medium for ~1 month to induce shoot development. Shoots 4–5 cm long were then cultured on rooting medium for ~2 weeks to induce root development and obtain T_0_ plants.

### Identification of Transgenic T_0_ Plants

Genomic DNA samples were extracted from T_0_ plants using the DNA-quick Plant System kit (Tiangen Biotech, Beijing, China). Target loci were amplified by PCR using specific xCas9 primers (Supplementary Table 2). Transgenic T_0_ plants were identified based on the amplification of an 854 bp fragment, which was detected by agarose gel electrophoresis.

### Mutant Identification

Several transgenic T_0_ plants were analyzed to identify gene mutations and C-to-T or A-to-G conversions. Target loci were amplified by PCR using specific primers (Supplementary Table 2). The PCR products were subjected to Sanger sequencing (General Biol, Anhui, China). The mutation types at the target sites (i.e., double peaks in the sequencing chromatograms) were determined using an online tool (http://skl.scau.edu.cn/dsdecode/) (Liu et al., 2015; Xie et al., 2017). The insertions/deletions (Indels) at or near the target sites were defined as gene mutations. Additionally, the frequency (%) of the C-to-T or A-to-G conversion was calculated based on the number of mutants with any target C-to-T or A-to-G substitution among all tested transgenic T_0_ plants. The frequency (%) of different mutation types was calculated based on the number of mutants with the same mutation type among all of the mutants. A mutant in which all of the C-to-T conversions at the target site were homozygous was considered to be a homozygous mutant.

## Results

### xCas9 Induces Gene Mutations at GAD (Where D is A, T, or G) PAM Sites in Rice Plants

**Figure 1:**
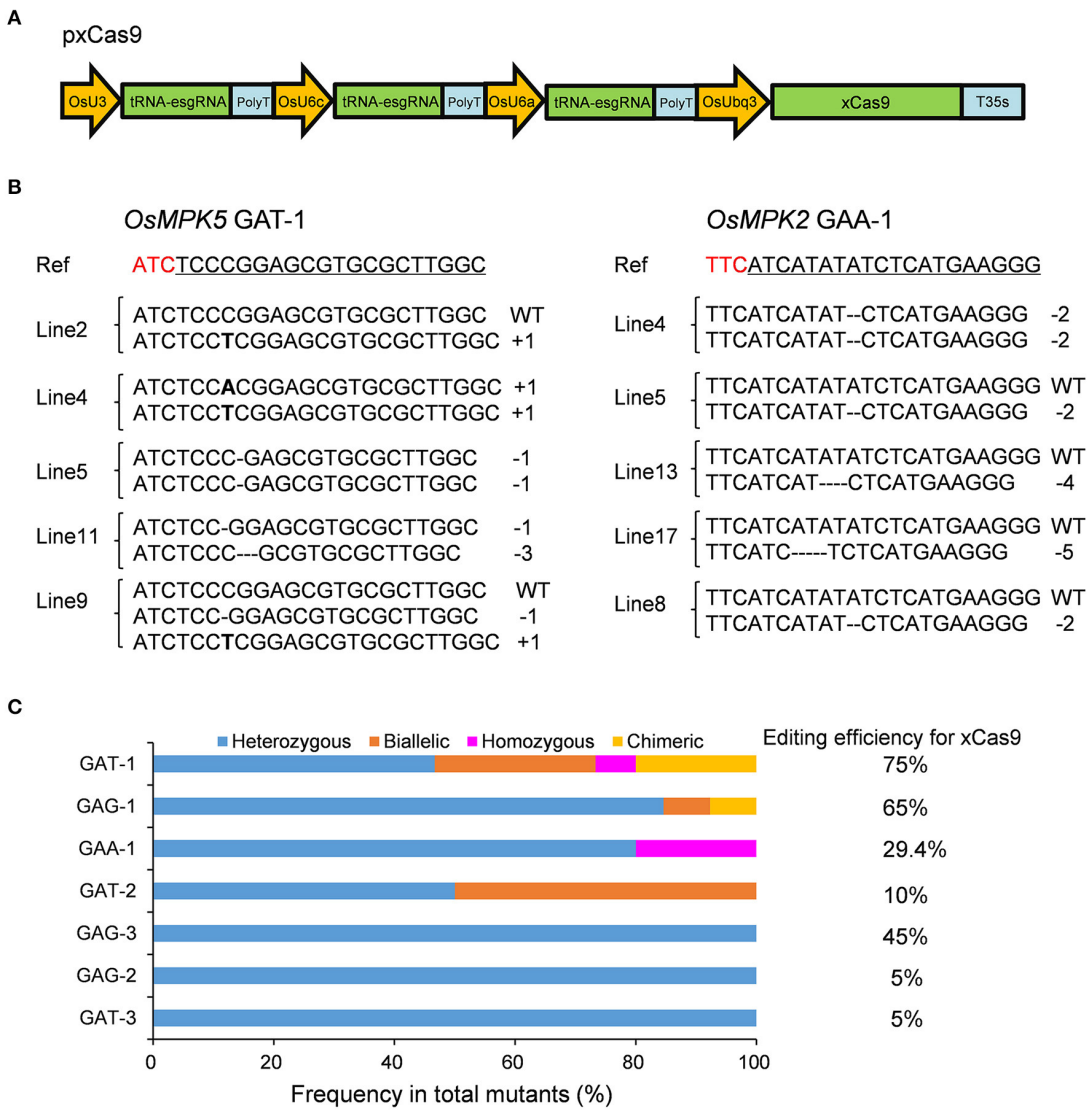
Gene mutagenesis at GAD PAM sites induced by xCas9 in rice T_0_ plants. **(A)** Schematic illustration of the pxCas9 construct for the CRISPR-xCas9 system. **(B)** Mutation types at GAA-1 and GAT-1 target sites of some representative stable mutant lines. Ref sequence is the wild-type sequence. Target sequences are underlined and PAM sequences are indicated in red. **(C)** Frequencies of different mutation types at GAD PAM sites mutated by xCas9.

In our previous study, we revealed that the tRNA-esgRNA system might help the xCas9-based CBE to efficiently edit target sites with a GA PAM in rice (Zhang et al., 2020). Therefore, we used tRNA and esgRNA to develop a CRISPR-xCas9 system to assess the cleavage activity of xCas9 in rice (Figure 1A). The rice codon-optimized xCas9 sequence with A262T/R324L/S409I/E480K/E543D/M694I/E1219V mutations (derived from SpCas9) (Zhang et al., 2020) under the control of the *Oryza sativa* ubiquitin (*OsUbq*) promoter was used in this study (Figure 1A). Each tRNA together with esgRNA was placed under the control of the rice *U3* or *U6* promoter (Figure 1A).

Because xCas9 can edit GAA and GAT PAM target sites in human cells (Hu et al., 2018), we first tested the editing activity of xCas9 in rice at three GAA PAM target sites in the *OsMPK2, OsMPK5*, and *OsNRT1.1B* genes (GAA-1, GAA-2, and GAA-3, respectively) (Supplementary Table 3) as well as at three GAT PAM sites in the *OsMPK5* or *OsWaxy* gene (GAT-1, GAT-2, and GAT-3) (Table 1 and Supplementary Table 3). In the T_0_ plants, all three GAT PAM sites were edited, with frequencies ranging from 5 to 75% (Figure 1B and Table 1). Of the three GAA PAM sites, only GAA-1 was edited, with a frequency of 29.4% (Figure 1B and Table 1). Accordingly, xCas9 can efficiently induce gene mutations at sites with GAA and GAT PAMs in rice.

**Table 1:** Summary of the mutation frequencies and mutation types at GA PAM sites targeted by xCas9.

**PAM sequence**	**Target site**	**Target gene**	**Target sequence**	**Tested T_**0**_ plants**	**Edited T_**0**_ plants**	**Mutation frequency (%)**	**Mutation types**
GAA	GAA-1	*OsMPK2*	CCCTTCATGAGATATATGATGAA	17	5	29.4	4He+1Ho
	GAA-2	*OsMPK5*	CACCTTCAACCCGCTGCAGAGAA	19	0	0	
	GAA-3	*OsNRT1.1B*	ACCAGGAGGTACAGGTTCAAGAA	18	0	0	
GAT	GAT-1	*OsMPK5*	GCCAAGCGCACGCTCCGGGAGAT	20	15	75	7He+4Bi+1Ho+3Chi
	GAT-2	*OsWaxy*	CTCATGCAGGAGGACGTCCAGAT	20	2	10	1He+1Bi
	GAT-3	*OsWaxy*	CTGCTCCTTGAAGAGCCTGAGAT	20	1	5	1He
GAG	GAG-1	*OsWaxy*	CGTCATTCCTGGAGAAGGTGGAG	20	13	65	11He+1Bi+1Chi
	GAG-2	*OsALS*	CCAACCACCTCTTCCGCCACGAG	20	1	5	1He
	GAG-3	*OsALS*	GCTTCCTCATGAACATTCAGGAG	20	9	45	9He
GAC	GAC-1	*OsWaxy*	TACCGAGGAGAGATCACCATGAC	20	0	0	
	GAC-2	*OsGRF4*	CACTTTCGTTCTTTGGGAACGAC	20	0	0	
	GAC-3	*OsNRT1.1B*	GCGACCACCATCATGTTCTGGAC	20	0	0	
	GAC-4	*OsALS*	ACCTTGTCCTTGATGTGGAGGAC	20	0	0	
	GAC-5	*OsWaxy*	CCACCGGCTTCGGCATCGCCGAC	20	0	0	
	GAC-6	*OsALS*	ACCTCGTGTCCGCGCTCGCCGAC	20	0	0	
	GAC-7	*OsNRT1.1B*	GCTTCGGCTCCGACCAGTTCGAC	20	0	0	
	GAC-8	*OsPDS*	TCCTGCAGAGGAATGGGTTGGAC	20	0	0	

*The PAM sequence is underlined. He, heterozygous mutation; Bi, biallelic mutation; Ho, homozygous mutation; Chi, chimeric mutation*.

Considering the ability of xCas9 to edit sites with GAA and GAT PAMs, we speculated that it might also be able to modify GAG and GAC PAM sites in rice. Thus, three target sites with GAG PAM sites in the *OsWaxy* or *OsALS* gene (GAG-1, GAG-2, and GAG-3) (Supplementary Table 3) and three sites with GAC PAM sites in the *OsWaxy, OsGRF4*, and *OsNRT1.1B* genes (GAC-1, GAC-2, and GAC-3, respectively), were analyzed (Table 1 and Supplementary Table 3). Although xCas9 edited all three targets with GAG PAM sites, with frequencies ranging from 5 to 65%, no mutations were detected at the three GAC PAM sites (Table 1). To further evaluate the editing capability of xCas9 at GAC PAM sites, we tested another five target sites (GAC-4 to -8), all of which were not mutated by xCas9 (Table 1). These results suggest that xCas9 can efficiently mutate target sites with GAG PAM sites.

These findings indicate that the CRISPR-xCas9 system is useful for expanding the potential editing sites in the rice genome to sequences including a GAD PAM. We subsequently characterized the mutation types generated by xCas9 by analyzing the sequencing chromatograms for the edited target sites. Four mutation types were identified, namely heterozygous, biallelic, homozygous, and chimeric (Figure 1C). Only heterozygous mutations were produced at GAT-3, GAG-2, and GAG-3 target sites, whereas two distinct mutation types were produced at GAT-2 and GAA-1 sites (Figure 1C). At two target sites that were efficiently edited, GAG-1 (65%) and GAT-1 (75%), three and four mutation types were identified, respectively (Figures 1B,C). We detected a high proportion of heterozygous mutations at all target sites. Additionally, the number of mutation types tended to increase as the editing efficiency increased.

Finally, to clarify the effect of the tRNA on the efficiency of the CRISPR-xCas9 system, we constructed a new vector without a tRNA sequence to target all three GAG PAM sites edited by xCas9 relatively efficiently. The resulting system mutated all three target sites less efficiently than the system with tRNA, with decreases in the editing frequency from 65 to 35% at the GAG-1 site, 5 to 0% at the GAG-2 site, and 45 to 38.9% at the GAG-3 site (Table 1 and Supplementary Table 4). These results imply that tRNA may not be required for the CRISPR-xCas9 system, but it increases the editing efficiency of the system.

### xCas9 Induces Gene Mutations at NG PAM Sites in Rice Plants

**Figure 2:**
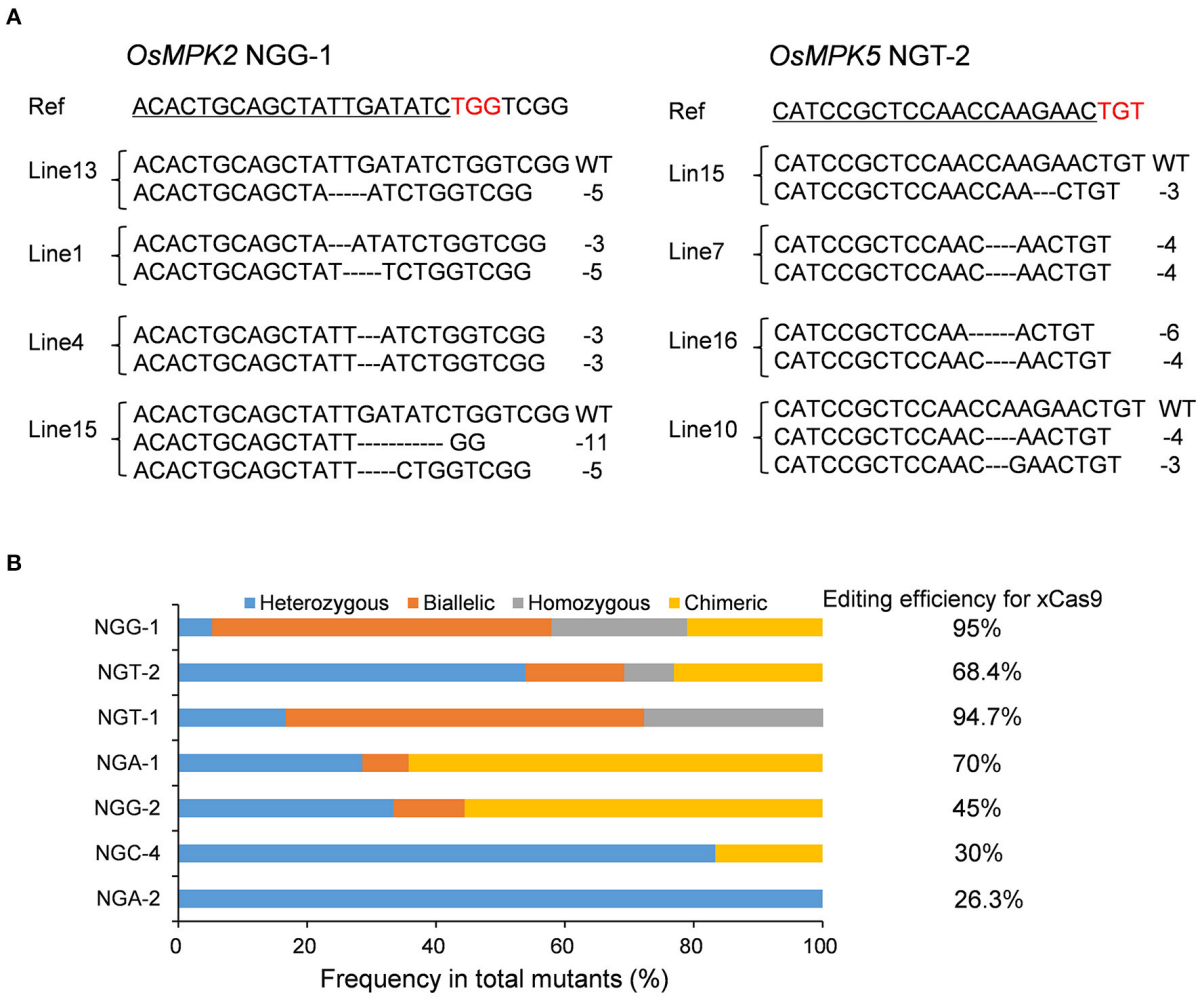
Gene mutagenesis at NG PAM sites induced by xCas9 in rice T_0_ plants. **(A)** Mutation types at NGG-1 and NGT-2 target sites of some representative stable mutant lines. Ref sequence is the wild-type sequence. Target sequences are underlined and PAM sequences are indicated in red. **(B)** Frequencies of different mutation types at NG PAM sites mutated by xCas9.

To assess whether the CRISPR-xCas9 system can edit target sites harboring NG PAMs, we tested two NGG, NGA, and NGT PAM sites and three NGC PAM sites (NGC-1, NGC-2, and NGC-3) in the *OsMPK2, OsMPK5, OsGRF4, OsNRT1.1B*, or *OsWaxy* genes (Table 2 and Supplementary Table 3). Analyses of rice T_0_ plants confirmed the robust editing activities of xCas9 at target sites with NGG, NGA, and NGT PAMs (Table 2). The editing efficiencies exceeded 90% at NGG-1 (95%) and NGT-1 (94.7%) (Figure 2A and Table 2). The NGA-1 and NGT-2 sites were also edited highly efficiently (~70%) (Figure 2A and Table 2). The NGG-2 site was also mutated, albeit less efficiently (45%) (Table 2). In contrast, the editing efficiency at NGA-2 was low (26.3%) (Table 2) and xCas9 did not modify any of the three NGC PAM sites. Another three NGC PAM sites in *OsMPK5, OsMPK2*, and *OsWaxy* were tested (NGC-4, NGC-5, and NGC-6, respectively) (Table 2 and Supplementary Table 3). Only the NGC-4 target site was mutated by xCas9, with an editing efficiency of 30% (Table 2). Collectively, these results suggest the CRISPR-xCas9 system can induce gene mutations at NGD PAM sites in the rice genome. It can also modify NGC PAM sites, but less efficiently. Moreover, on average, xCas9 can edit NG PAM sites more efficiently than GAD PAM sites.

**Table 2:** Summary of the mutation frequencies and mutation types at NG PAM sites targeted by xCas9.

**PAM sequence**	**Target site**	**Target gene**	**Target sequence**	**Tested T_**0**_ plants**	**Edited T_**0**_ plants**	**Mutation frequency (%)**	**Mutation types**
NGG	NGG-1	*OsMPK2*	ACACTGCAGCTATTGATATCTGG	20	19	95	1He+10Bi+4Ho+4Chi
	NGG-2	*OsMPK5*	CGACATGATGACGGAGTACGTGG	20	9	45	3He+1Bi+5Chi
NGA	NGA-1	*OsGRF4*	GCATTCTCATCAGCGAGGTCTGA	20	14	70	4He+1Bi+9Chi
	NGA-2	*OsNRT1.1B*	GCTCTACCTGGGGCTCTACCTGA	19	5	26.3	5He
NGT	NGT-1	*OsMPK2*	CAACGCCCGCAGATATGTGAGGT	19	18	94.7	3He+10Bi+5Ho
	NGT-2	*OsMPK5*	CATCCGCTCCAACCAAGAACTGT	19	13	68.4	7He+2Bi+1Ho +3Chi
NGC	NGC-1	*OsMPK5*	TCAGGCCGACGATGACGCACGGC	20	0	0	
	NGC-2	*OsMPK2*	AGACCTCAGGCCAAGTAATTTGC	20	0	0	
	NGC-3	*OsWaxy*	GGCACACTGGCCCACTGGCGAGC	20	0	0	
	NGC-4	*OsMPK5*	AGCCGCCCATCATGCCCATTGGC	20	6	30	5He+1Chi
	NGC-5	*OsMPK2*	CCACCTTCTTCGATCAAACCAGC	20	0	0	
	NGC-6	*OsWaxy*	TCGGCCACCGGCTTCGGCATCGC	20	0	0	

*The PAM sequence is underlined. He, heterozygous mutation; Bi, biallelic mutation; Ho, homozygous mutation; Chi, chimeric mutation*.

A subsequent examination identified four mutation types generated by xCas9 at the above edited NG PAM target sites (Figure 2B). Only heterozygous mutations were detected at the NGA-2 site (Figure 2B), in contrast to the heterozygous and chimeric mutations at the NGC-4 site (Figure 2B). Three or four mutation types were detected at the other five sites that were edited relatively efficiently (Figure 2B). We observed that heterozygous mutations could be generated at all analyzed target sites. Furthermore, the number of mutation types generally increased as the editing efficiency increased, which was consistent with the results for the GAD PAM sites. However, unlike the GAD PAM sites, heterozygous mutations were not the predominant mutation at all target sites. The ratio of chimeric or biallelic mutations was relatively high, and these mutations accounted for a large proportion of the modifications at the NGG-2, NGA-1, NGT-1, and NGG-1 sites (Figure 2B).

### Base Editing by xCas9 in Rice

**Figure 3:**
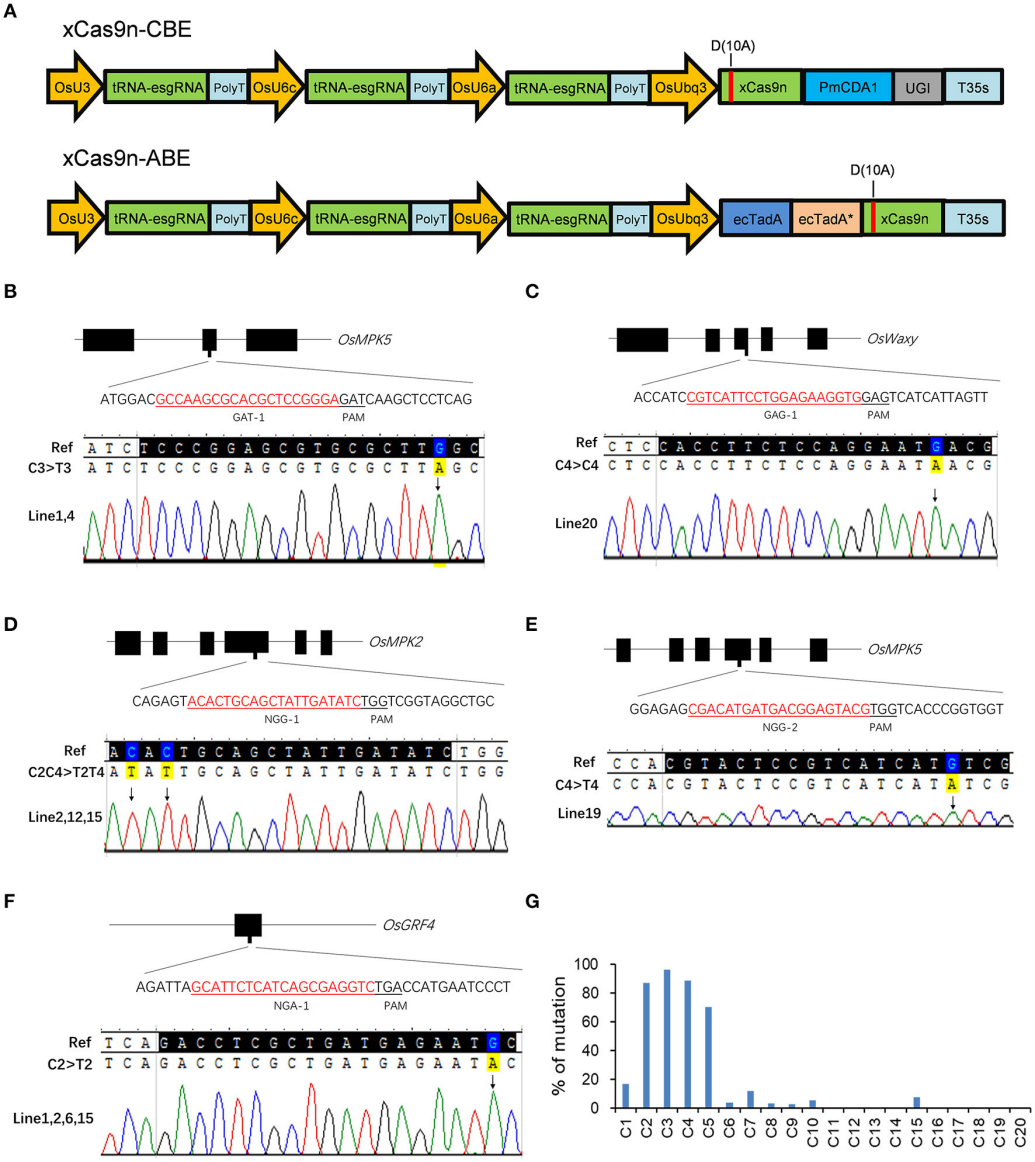
Base editing by xCas9 in rice T_0_ plants. **(A)** Schematic view of xCas9n-CBE and xCas9n-ABE. **(B–F)** Sequencing chromatograms of all homozygous mutant lines in which xCas9n-CBE modified the GAT-1 **(B)**, GAG-1 **(C)**, NGG-1 **(D)**, NGG-2 **(E)**, and NGA-1 **(F)** target sites. The mutated bases are marked by arrows. **(G)** Ratio of T_0_ plants mutated at a certain position to all genome-edited T_0_ plants containing C at that position. The x-axis presents the position of C from the 5′ end of the target.

On the basis of the efficient editing of the GAD and NG PAMs by the CRISPR-xCas9 system, we decided to develop a new xCas9-based CBE and ABE using vectors that were structurally similar to those used to produce the CRISPR-xCas9 system for editing the bases at the same PAM sites in rice. First, xCas9 was mutated to xCas9(D10A) nickase (xCas9n) via PCR and then fused to *Petromyzon marinus* cytidine deaminase1 (PmCDA1) and uracil DNA glycosylase inhibitor (UGI) to generate xCas9n-CBE (Figure 3A). The 18 target sites with GAA, GAT, GAG, NGG, NGA, NGT, and NGC sequences used for testing the CRISPR-xCas9 system were selected to evaluate the C-to-T base editing activities of xCas9n-CBE (Table 3).

**Table 3:** C-to-T mutations induced by xCas9n-CBE in rice T_0_ plants.

**PAM sequence**	**Target site**	**Tested T_**0**_ plants**	**Edited T_**0**_ plants**	**C-to-T frequency (%)**	**Genotypes**	**Homozygous**
GAA	GAA-1	12	4	33.3	C2>T2(1); C2C3>T2T3(1); C1C2C3>T1T2T3(2)	0
	GAA-2	12	0	0		
	GAA-3	12	0	0		
GAT	GAT-1	19	9	47.4	C3>T3(3); C2C3>T2T3(6)	2
	GAT-2	20	0	0		
	GAT-3	20	0	0		
GAG	GAG-1	20	14	70	C4>T4(10); C1C4>T1T4(3); C4C9>T4T9(1)	2
	GAG-2	20	2	10	C15>T15(2)	0
	GAG-3	20	4	20	C2>T2(2); C2C5>T2T5(2)	0
NGG	NGG-1	19	13	68.4	C2>T2(1); C2C4>T2T4(10); C2C4C7>T2T4T7(2)	3
	NGG-2	20	5	25	C4>T4(5)	1
NGA	NGA-1	20	13	65	C2>T2(12); C2C6C8>T2T6T8(1)	4
	NGA-2	19	1	5.3	C4>T4(1)	0
NGT	NGT-1	18	12	66.7	C1>T1(1); C4>T4(5); C7>T7(2); C1C4>T1T4(1); C4C7>T4T7(1); C6C7>T6T7(1); C1C7C10>T1T7T10(1)	0
	NGT-2	20	10	50	C5>T5(1); C4C5>T4T5(8); C4C5C10>T4T5T10(1)	0
NGC	NGC-4	27	14	51.9	C3C4>T3T4(13); C3C4C8>T3T4T8(1)	0
	NGC-5	29	0	0		
	NGC-6	30	0	0		
GAC	GAC-1	19	2	10.5	C3>T3(1); C3C4>T3T4(1)	0
	GAC-2	20	0	0		
	GAC-3	20	1	5	C2>T2(1)	0

As expected, xCas9n-CBE efficiently mediated the C-to-T base conversion at the tested target sites (Table 3). An examination of the T_0_ plants indicated that of the three GAA PAM sites, GAA-1 was edited, with a frequency of 33.3%, whereas GAA-2 and GAA-3 were not modified (Table 3), which was consistent with the editing results for the CRISPR-xCas9 system. Among the three target sites with the GAT PAM, only GAT-1 was edited by xCas9n-CBE, with a mutation rate of 47.4% (Figure 3B and Table 3). Base mutations were undetectable at GAT-2 and GAT-3, although Indel mutations were induced by xCas9 (Tables 1, 3). Additionally, xCas9n-CBE edited all three targets with GAG PAMs, with frequencies ranging from 10 to 70% (Figure 3C and Table 3). For the nine NG PAM sites, xCas9n-CBE induced C-to-T substitutions at all target sites that were edited by the CRISPR-xCas9 system (Figures 3D–F, Tables 1,3).

These results imply that xCas9n-CBE and the CRISPR-xCas9 system are two distinct editing systems, with some differences in the editable target sites. Therefore, although xCas9 inefficiently edits GAC PAM sites, because we previously confirmed that the tRNA-esgRNA system (xCas9n-epBE) can efficiently edit these PAM sites (Zhang et al., 2020), we chose the same target sites (GAC-1, GAC-2, and GAC-3) to determine whether xCas9n-CBE can modify GAC PAM sites. Surprisingly, base mutations were detected at GAC-1 and GAC-3, with frequencies of 10.5 and 5%, respectively, whereas no base mutations were detected at GAC-2 (Table 3). These findings suggest that xCas9n-CBE can efficiently mediate the C-to-T base conversion even at target sites with GAC PAMs, albeit with a relatively low editing efficiency.

Considered together, our results indicate that xCas9n-CBE induces the C-to-T base conversion at GA and NG PAM sites in rice. Thus, it can target a wider range of sequences than the CRISPR-xCas9 system, which inefficiently edits GAC PAM sites. To further characterize xCas9n-CBE regarding its editing window, editing preference, and mutation types, all 104 genome-edited T_0_ plants for the above-mentioned 14 edited target sites were analyzed together. The editing window typically spanned positions 1 to 10 within the protospacer and occasionally extended to position 15 (Figure 3G). In the editing window, C2, C3, C4, and C5 were edited more frequently than C1 and C7 (C3 > C2 ≈ C4 > C5 >> C1 ≈ C7), whereas C6, C8, C9, C10, and C15 were mutated relatively infrequently (<10%) (Figure 3G). In the T_0_ plants, single- and double-base substitutions were the predominant mutations at the edited targets. Single C-to-T mutations were detected at almost all edited sites, and no other types of base substitutions were detected at three target sites with a mutation frequency of 10% or less (Table 3). Almost all of the triple-base substitutions were detected at targets with an editing efficiency of 50% or more (Figure 3G). Furthermore, five of the 14 edited target sites had homozygous mutations (Figures 3B–F, Table 3).

We also fused the wild-type adenine deaminase ecTadA and its variant ecTadA^*^ to the N-terminus of xCas9n to generate xCas9n-ABE in rice (Figure 3A). All 21 target sites used to test xCas9n-CBE were also used for assessing the editing capability of xCas9n-ABE. Unfortunately, the sequencing results revealed a lack of A-to-G mutations in the T_0_ rice plants (data not shown).

## Discussion

In this study, the utility of xCas9 for targeted gene mutation was thoroughly investigated by analyzing its editing activities at several endogenous target sites with GA and NG PAMs in rice. The analyses revealed that xCas9 can recognize a broad range of PAM sequences, including NG, GAA, and GAT, which is consistent with the results of a recent study involving human cells (Hu et al., 2018). Additionally, xCas9 can also induce Indel mutations at GAG PAM sites, which have not been observed in human cells. Although there have been many studies on the editing activities of xCas9 in rice, no edited target sites with GAA and GAG PAM sequences have been detected in T_0_ plants (Hua et al., 2019; Li et al., 2019; Ren et al., 2019; Wang et al., 2019a; Zhong et al., 2019; Zeng et al., 2020). Therefore, the CRISPR-xCas9 system developed in the current study will be useful for expanding the range of potential gene mutations by targeting these PAM sites in rice.

We used tRNA and esgRNA in our CRISPR-xCas9 system. We determined that tRNA increased the editing efficiency of xCas9 (Table 1 and Supplementary Table 4), which was consistent with the results of earlier studies in which the tRNA-sgRNA system enhanced the activity of high-fidelity Cas9 variants in human cells and rice (Zhang et al., 2017; He et al., 2019). Our previous research proved that the tRNA-esgRNA system enables xCas9n-epBE to efficiently edit GA and NG PAM sites (Zhang et al., 2020). We speculated that tRNA-esgRNA is also important for facilitating efficient gene mutations via the CRISPR-xCas9 system.

The xCas9n-CBE system described herein differs from our previously reported xCas9n-epBE system in terms of the associated vector architecture (Zhang et al., 2020). They belong to different multiplex editing systems. Three independent tRNA-esgRNAs under the control of three different promoters were employed in xCas9n-CBE, whereas one or more tRNA-esgRNAs in xCas9n-epBE were controlled by a common promoter. Both xCas9n-CBE and xCas9n-epBE can efficiently modify bases at GA and NGD (where D is G, T, or A) PAM sites. However, xCas9n-CBE can efficiently edit NGC PAM sites, whereas xCas9n-epBE cannot. Additionally, on average, xCas9n-CBE can edit NG PAM sites more efficiently than xCas9n-epBE (Table 2). Moreover, xCas9n-CBE has a wider editing window than xCas9n-epBE because it can target C8, C9, and C15 (Figure 3G). Thus, xCas9n-CBE might be a better alternative to xCas9n-epBE.

Regrettably, the adenine base editor (xCas9n-ABE) developed in our study failed to modify the tested target sites, which was in contrast to the results of a recent study involving human cells (Hu et al., 2018). Although Hua et al. (2019) identified one NGT PAM site edited by ABE-P6, the editing efficiency was quite low (4.8%) and none of the other five tested PAM sites were edited. Additionally, no target sites were edited by the xCas9-based ABE in rice in another previous study (Zeng et al., 2020). Considering that the editing efficiency of xCas9 at the same target sites contrasted with the relative lack of editing by the xCas9-based ABE, the editing activity of xCas9n-ABE in rice will need to be improved in future studies.

Because some of the target sites were edited relatively inefficiently by the CRISPR-xCas9 system or xCas9n-CBE, further research applying highly efficient nuclear localization signals and surrogate systems is required to increase the editing efficiency (Wang et al., 2019b, 2020; Xu et al., 2020). Only one of six NGC PAM sites was successfully mutated by xCas9 (Table 2). This is similar to the results reported by Hua et al. (2019). Moreover, SpCas9-NG also had limited activity at NGC PAM sites in human cells and rice (Nishimasu et al., 2018; Ren et al., 2019). Accordingly, the editing efficiency at NGC PAM sites will need to be increased. Three new SpCas9 variants, SpCas9-NRRH, SpCas9-NRTH, and SpCas9-NRCH, were recently reported to recognize non-G PAMs in human cells (Miller et al., 2020). Furthermore, a near PAM-less SpCas9 variant (SpRY) was also developed (Walton et al., 2020). The utility of these new Cas9 variants should be tested in rice to further expand the genome editing scope.

## Conclusion

In this study, we developed an efficient CRISPR-xCas9 system that can expand the potential target genome sequences to include GAD PAM sites in rice. It can also mutate genes at NG PAM sites. We also developed xCas9n-CBE with a similar vector architecture. The efficient base-editing activities of xCas9n-CBE at GA and NG PAM sites were confirmed, with the main deamination window comprising protospacer positions 1 to 10. These new genome engineering tools will be useful for future basic rice research and crop improvement.

## Data Availability Statement

The raw data supporting the conclusions of this article will be made available by the authors, without undue reservation.

## Author Contributions

JY and CZ designed the experiments. XL, SZ, SY, LL, YY, and FW performed all the experiments. CZ, GK, and XZ analyzed the results. CZ and JY wrote the manuscript. JY supervised the project. All authors read and approved the final manuscript.

## Conflict of Interest

The authors submitted patent applications based on the results reported in this paper.
