# Supplementary material for: Genome Engineering in Plant Using an Efficient CRISPR-xCas9 Toolset With an Expanded PAM Compatibility
Source: Front Genome Ed. 2020 Dec 16;2:618385. doi: 10.3389/fgeed.2020.618385 (PMC8525348; doi:10.3389/fgeed.2020.618385)
Supplement: Supplementary file 1 [file Table_1.DOCX]

Supplementary Material

Genome engineering in plant using an efficient CRISPR-xCas9 toolset with an expanded PAM compatibility

**Chengwei Zhang, Guiting Kang, Xinxiang Liu, Si Zhao, Shuang Yuan, Lu Li, Yongxing Yang, Feipeng Wang, Xiang Zhang and Jinxiao Yang***

*** Correspondence:**

Dr. Jinxiao Yang

yangjinxiao@maizedna.org

# Supplementary Data

**xCas9 sequence**

atggactacaaggaccacgacggggattacaaagaccacgacatagactacaaggatgacgatgacaaaatggcaccgaagaaaaaaaggaaggtcggaatccatggcgttccagctgccgataagaaatattccatcggactcgacattggcacgaatagcgtcggatgggctgttattactgatgagtacaaagttccgtctaagaagttcaaggtgctgggcaacacagaccgccacagcataaagaaaaatctcatcggtgcactccttttcgatagtggggagactgcagaagcgacaagattgaaaaggactgcgagaaggcgctatacacggcgtaagaatagaatctgctaccttcaggagattttctctaacgaaatggctaaggtcgatgacagtttctttcatagacttgaggaatcgttcttggttgaggaggataagaaacatgagaggcacccgatatttggaaacatcgtggatgaggtcgcatatcatgaaaagtaccccacaatctaccacctgagaaagaaactcgttgattccaccgacaaagcggatttgagactcatctacctcgctcttgcccatatgataaagttccgcggacactttctgatcgagggcgacctcaaccctgataatagcgacgtcgataagctcttcatccagttggttcaaacctacaatcagctctttgaggaaaacccaattaatgctagtggagtggatgcaaaagcgatactgtcggccagactctccaagagcagaaggttggagaacctgatcgctcaacttcctggagaaaagaaaaacggtctttttgggaatttgattgccttgtctctgggcctcacaccaaacttcaagtcaaattttgacctcgctgaggataccaaacttcagttgtctaaggatacctatgatgacgatcttgacaatttgctggcacaaattggcgaccagtacgcggatctgttcctcgcagcgaagaatctgagtgatgctattctcctttcggacatactcagggttaacactgagatcacaaaagcacctttgagtgcgtcgatgattaagctgtatgatgaacatcaccaagacctcactttgctgaaggcccttgtgcggcagcaattgccagagaagtacaaagaaatcttctttgaccaatctaagaacggatacgctggctatattgatggaggagcttctcaggaggaattctataagtttatcaaacctatacttgagaagatggatggtacagaggaactccttgttaaattgaacagagaagatttgctgcgcaagcaacggacctttgacaacggaatcattccgcatcagatacacctcggcgagcttcatgccatccttcgccggcaggaagatttctacccctttttgaaggacaaccgcgagaagatagaaaaaatccttacgttccggattccttactatgtgggtccattggcaagggggaattcccgctttgcgtggatgactcggaaaagcgaggaaactatcacaccgtggaacttcgagaaagttgtggacaagggagcttctgcccaatcattcattgagaggatgactaacttcgataagaacctgccgaacgagaaagttctccccaagcactccctcctttacgagtatttcaccgtgtataacgaacttacgaaggttaaatacgtgactgagggtatgaggaagccagcattcttgagcggggatcaaaagaaagcgattgttgatttgctgtttaaaactaatcgcaaggtgacagtcaagcagctcaaagaggattatttcaagaaaattgaatgtttcgactctgtggagatatcaggagtcgaagataggtttaacgcttcccttggcacataccatgacctccttaagatcattaaggacaaagatttcctggataacgaggaaaatgaggacatcctcgaagatattgttcttaccttgacgctgtttgaggatcgcgaaatgatcgaggaacggcttaagacgtatgctcacttgttcgacgataaggttatgaagcagctcaagcgtagaaggtacactggatggggccgtctgtctagaaagctcatcaacggaatacgtgataaacaaagtggcaagacaattttggattttctgaagtcggacggattcgccaacagaaattttattcagctgattcatgacgatagtctcaccttcaaagaggacatacagaaggctcaagtgagtggtcaaggggattcgctgcatgaacacatcgcaaacctcgcgggttcaccggccataaagaaaggaatccttcaaactgttaaggtcgttgatgagttggttaaagtgatgggtaggcacaagcccgaaaacatagtgatcgagatggctcgcgaaaatcagactacacaaaaagggcagaagaactctcgcgagcggatgaaaaggattgaggaaggaatcaaggaactgggctcacagattctcaaagagcatccagtcgaaaacacacagctgcaaaatgagaagctctatctttactatctccaaaatggccgggacatgtatgttgatcaggagcttgacatcaaccgtttgtccgactatgatgtggaccacattgtcccgcaatctttccttaaggacgattcaatcgataataaggtgttgacccggagcgataaaaaccgtggaaagtctgacaatgtcccttcagaggaagtggttaagaagatgaagaactactggagacaattgctgaatgcaaaactgatcacacagagaaagttcgacaacctcaccaaagcagagagaggtgggctcagtgaacttgataaagcgggcttcattaagcgtcagctcgttgagactagacagatcacgaagcatgtcgcgcagattttggattcgcggatgaacacgaagtacgacgagaatgataaactgatacgtgaagtcaaggttatcactcttaagtccaaattggtgagcgatttcagaaaggacttccaattctataaggtcagggagatcaacaattatcatcacgctcacgatgcctaccttaatgctgttgtggggaccgcccttattaagaaataccctaaattggagtctgaattcgtttacggggattataaggtctacgacgttaggaaaatgatagctaagagtgagcaggagatcggtaaagcaactgcgaagtatttcttttactcgaacatcatgaatttctttaagaccgagataacgctggcaaatggcgaaattagaaagaggcctctcatagagactaacggtgagacaggggaaatcgtctgggataagggtagggactttgcgacagtgcgcaaggtcctctctatgccgcaagttaatattgtgaagaaaaccgaggtgcagacgggaggcttctccaaggaaagcatacttcccaaacggaactctgataagttgatcgctcgtaagaaagattgggaccctaagaaatatggtgggttcgattccccaactgttgcttacagcgtgctggtcgttgccaaggtcgagaagggtaaatccaagaaactcaaaagcgttaaggaactccttgggattactatcatggagagatcttcattcgaaaagaatcctatcgactttcttgaggccaaaggatataaggaagttaagaaagatctgataatcaaactcccaaagtactcattgtttgagctggaaaacggcaggaagcgcatgcttgcttccgccggagttttgcagaaagggaacgagttggctctgccttctaagtatgttaacttcctctatcttgcctctcattacgagaagctcaaaggctcaccagaggacaacgaacagaaacaactttttgtcgagcaacataagcactatttggatgagattatagaacagatcagtgaattctcgaaaagggttatccttgcagatgcgaatcttgacaaggtgttgtctgcatacaacaaacatagagataagccgatcagggagcaagcggaaaatatcattcacctcttcactcttacaaacttgggtgctcccgctgccttcaagtattttgataccacgattgaccggaaacgttacacctcaacgaaggaggtgctggatgccaccctcatccaccaatctattaccggactctacgagactagaatcgatctctcacagctcggcggggataaaagaccagcagcgacgaaaaaggcaggacaggctaagaagaagaaatag

# Supplementary Tables

**Supplementary Table S1 |** Primers used in for plasmid construction.

| Primer | Sequence (5’-3’) | Purpose | Target site |
| --- | --- | --- | --- |
| xCas9-GAA-F1 | TATCGGATCCAACAAAGCACCAGTGGTCTAG | Construction of xCas9-GAA | for GAA-1 |
| xCas9-GAA-R1 | TATCGGTCTCAAAACATCATATATCTCATGAAGGGTGCACCAGCCGGGAA |  |  |
| xCas9-GAA-F2 | TATCGGTCTCAGTTTCAGAGCTATGCTGG |  |  |
| xCas9-GAA-R2 | TATCGGTCTCATGCACCAGCCGGGAA |  |  |
| xCas9-GAA-F3 | TATCGGTCTCATGCACACCTTCAACCCGCTGCAGAGTTTCAGAGCTATGCTGG |  | for GAA-2 |
| xCas9-GAA-R3 | TATCGGTCTCATGGTTGCACCAGCCGGGAA |  |  |
| xCas9-GAA-F4 | TATCGGTCTCAACCAGGAGGTACAGGTTCAAGTTTCAGAGCTATGCTGG |  | for GAA-3 |
| xCas9-GAA-R4 | CTTGACCCGAATTTGTAAGC |  |  |
| xCas9-GAT-F1 | TATCGGATCCAACAAAGCACCAGTGGTCTAG | Construction of xCas9-GAT | for GAT-1 |
| xCas9-GAT-R1 | TATCGGTCTCAAAACTCCCGGAGCGTGCGCTTGGCTGCACCAGCCGGGAA |  |  |
| xCas9-GAT-F2 | TATCGGTCTCAGTTTCAGAGCTATGCTGG |  |  |
| xCas9-GAT-R2 | TATCGGTCTCATGCACCAGCCGGGAA |  |  |
| xCas9-GAT-F3 | TATCGGTCTCATGCACTCATGCAGGAGGACGTCCAGTTTCAGAGCTATGCTGG |  | for GAT-2 |
| xCas9-GAT-R3 | TATCGGTCTCAGCAGTGCACCAGCCGGGAA |  |  |
| xCas9-GAT-F4 | TATCGGTCTCACTGCTCCTTGAAGAGCCTGAGTTTCAGAGCTATGCTGG |  | for GAT-3 |
| xCas9-GAT-R4 | CTTGACCCGAATTTGTAAGC |  |  |
| xCas9-GAG-F1 | TATCGGATCCAACAAAGCACCAGTGGTCTAG | Construction of xCas9-GAG | for GAG-1 |
| xCas9-GAG-R1 | TATCGGTCTCAAAACCACCTTCTCCAGGAATGACGTGCACCAGCCGGGAA |  |  |
| xCas9-GAG-F2 | TATCGGTCTCAGTTTCAGAGCTATGCTGG |  |  |
| xCas9-GAG-R2 | TATCGGTCTCATGCACCAGCCGGGAA |  |  |
| xCas9-GAG-F3 | TATCGGTCTCATGCACCAACCACCTCTTCCGCCACGTTTCAGAGCTATGCTGG |  | for GAG-2 |
| xCas9-GAG-R3 | TATCGGTCTCAAAGCTGCACCAGCCGGGAA |  |  |
| xCas9-GAG-F4 | TATCGGTCTCAGCTTCCTCATGAACATTCAGGTTTCAGAGCTATGCTGG |  | for GAG-3 |
| xCas9-GAG-R4 | CTTGACCCGAATTTGTAAGC |  |  |
| xCas9-GAC-F1 | TATCGGATCCAACAAAGCACCAGTGGTCTAG | Construction of xCas9-GAC-1 | for GAC-1 |
| xCas9-GAC-R1 | TATCGGTCTCAAAACATGGTGATCTCTCCTCGGTATGCACCAGCCGGGAA |  |  |
| xCas9-GAC-F2 | TATCGGTCTCAGTTTCAGAGCTATGCTGG |  |  |
| xCas9-GAC-R2 | TATCGGTCTCATGCACCAGCCGGGAA |  |  |
| xCas9-GAC-F3 | TATCGGTCTCATGCACACTTTCGTTCTTTGGGAACGTTTCAGAGCTATGCTGG |  | for GAC-2 |
| xCas9-GAC-R3 | TATCGGTCTCATCGCTGCACCAGCCGGGAA |  |  |
| xCas9-GAC-F4 | TATCGGTCTCAGCGACCACCATCATGTTCTGGTTTCAGAGCTATGCTGG |  | for GAC-3 |
| xCas9-GAC-R4 | CTTGACCCGAATTTGTAAGC |  |  |
| xCas9-GAC-F5 | ACTATCGGATCCAACAAAGCACCAGTGGTCTAGTGG | Construction of xCas9-GAC-2 | for GAC-4 |
| xCas9-GAC-R5 | ACTATCGGTCTCAAAACCTCCACATCAAGGACAAGGTTGCACCAGCCGGGAATCGAACC |  |  |
| xCas9-GAC-F6 | ACTATCGGTCTCAGTTTCAGAGCTATGCTGGAAACAG |  |  |
| xCas9-GAC-R6 | ACTATCGGTCTCATGCACCAGCCGGGAATCGAACCC |  |  |
| xCas9-GAC-F7 | ACTATCGGTCTCATGCAGCGACCACCATCATGTTCTGGTTTCAGAGCTATGCTGGAAAC |  |  |
| xCas9-GAC-R7 | ACTATCGGTCTCAGTGGTGCACCAGCCGGGAATCGAACCC |  |  |
| xCas9-GAC-F8 | ACTATCGGTCTCACCACCGGCTTCGGCATCGCCGTTTCAGAGCTATGCTGGAAACAG |  | for GAC-5 |
| xCas9-GAC-R8 | ATTTGTAAGCTTAAAAAAAAAAAAGCACCGACTCGGTGCCACTTTTTC |  |  |
| xCas9-GAC-F9 | ACTATCGGATCCAACAAAGCACCAGTGGTCTAGTGG | Construction of xCas9-GAC-3 | for GAC-6 |
| xCas9-GAC-R9 | ACTATCGGTCTCAAAACGGCGAGCGCGGACACGAGGTTGCACCAGCCGGGAATCGAAC |  |  |
| xCas9-GAC-F10 | ACTATCGGTCTCAGTTTCAGAGCTATGCTGGAAACAG |  |  |
| xCas9-GAC-R10 | ACTATCGGTCTCATGCACCAGCCGGGAATCGAACCC |  |  |
| xCas9-GAC-F11 | ACTATCGGTCTCATGCAGCTTCGGCTCCGACCAGTTCGTTTCAGAGCTATGCTGGAAAC |  | for GAC-7 |
| xCas9-GAC-R11 | ACTATCGGTCTCAAGGATGCACCAGCCGGGAATCGAACCC |  |  |
| xCas9-GAC-F12 | ATATCGGTCTCATCCTGCAGAGGAATGGGTTGGTTTCAGAGCTATGCTGGAAACAG |  | for GAC-8 |
| xCas9-GAC-R12 | ATTTGTAAGCTTAAAAAAAAAAAAGCACCGACTCGGTGCCACTTTTTC |  |  |
| xCas9-NGG&NGA-F1 | TATCGGATCCAACAAAGCACCAGTGGTCTAG | Construction of xCas9-NGG&NGA | for NGG-1 |
| xCas9-NGG&NGA-R1 | TATCGGTCTCAAAACGATATCAATAGCTGCAGTGTTGCACCAGCCGGGAA |  |  |
| xCas9-NGG&NGA-F2 | TATCGGTCTCAGTTTCAGAGCTATGCTGG |  |  |
| xCas9-NGG&NGA-R2 | TATCGGTCTCATGCACCAGCCGGGAA |  |  |
| xCas9-NGG&NGA-F3 | TATCGGTCTCATGCACGACATGATGACGGAGTACGGTTTCAGAGCTATGCTGG |  | for NGG-2 |
| xCas9-NGG&NGA-R3 | TATCGGTCTCAATGCTGCACCAGCCGGGAA |  |  |
| xCas9-NGG&NGA-F4 | TATCGGTCTCAGCATTCTCATCAGCGAGGTCGTTTCAGAGCTATGCTGG |  | for NGA-1 |
| xCas9-NGG&NGA-R4 | CTTGACCCGAATTTGTAAGC |  |  |
| xCas9-NGA&NGT-F5 | TATCGGATCCAACAAAGCACCAGTGGTCTAG | Construction of xCas9-NGA&NGT | for NGT-1 |
| xCas9-NGA&NGT-R5 | TATCGGTCTCAAAACTCACATATCTGCGGGCGTTGTGCACCAGCCGGGAA |  |  |
| xCas9-NGA&NGT-F6 | TATCGGTCTCAGTTTCAGAGCTATGCTGG |  |  |
| xCas9-NGA&NGT-R6 | TATCGGTCTCATGCACCAGCCGGGAA |  |  |
| xCas9-NGA&NGT-F7 | TATCGGTCTCATGCACATCCGCTCCAACCAAGAACGTTTCAGAGCTATGCTGG |  | for NGT-2 |
| xCas9-NGA&NGT-R7 | TATCGGTCTCAGAGCTGCACCAGCCGGGAA |  |  |
| xCas9-NGA&NGT-F8 | TATCGGTCTCAGCTCTACCTGGGGCTCTACCGTTTCAGAGCTATGCTGG |  | for NGA-2 |
| xCas9-NGA&NGT-R8 | CTTGACCCGAATTTGTAAGC |  |  |
| xCas9-NGC-F1 | ACAACTGGATCCAACAAAGCACCAGTGGTCTAGTGG | Construction of xCas9-NGC-1 | for NGC-1 |
| xCas9-NGC-R1 | ACTACAGGTCTCAAAACGTGCGTCATCGTCGGCCTGATGCACCAGCCGGGAATCGAACC |  |  |
| xCas9-NGC-F2 | ACAACTGGTCTCAGTTTCAGAGCTATGCTGGAAACAG |  |  |
| xCas9-NGC-R2 | TATCGTGGTCTCATGCACCAGCCGGGAATCGAACCC |  |  |
| xCas9-NGC-F3 | ACTATCGGTCTCATGCAAGACCTCAGGCCAAGTAATTGTTTCAGAGCTATGCTGGAAAC |  | for NGC-2 |
| xCas9-NGC-R3 | TATCGTGGTCTCATGCCTGCACCAGCCGGGAATCGAACCC |  |  |
| xCas9-NGC-F4 | ACTATCGGTCTCAGGCACACTGGCCCACTGGCGGTTTCAGAGCTATGCTGGAAACAG |  | for NGC-3 |
| xCas9-NGC-R4 | ATTTGTAAGCTTAAAAAAAAAAAAGCACCGACTCGGTGCCACTTTTTC |  |  |
| xCas9-NGC-F5 | ACTATCGGATCCAACAAAGCACCAGTGGTCTAGTGG | Construction of xCas9-NGC-2 | for NGC-4 |
| xCas9-NGC-R5 | ACTATCGGTCTCAAAACAATGGGCATGATGGGCGGCTTGCACCAGCCGGGAATCGAACC |  |  |
| xCas9-NGC-F6 | ACTATCGGTCTCAGTTTCAGAGCTATGCTGGAAACAG |  |  |
| xCas9-NGC-R6 | ACTATCGGTCTCATGCACCAGCCGGGAATCGAACCC |  |  |
| xCas9-NGC-F7 | ACTATCGGTCTCATGCACCACCTTCTTCGATCAAACCGTTTCAGAGCTATGCTGGAAAC |  | for NGC-5 |
| xCas9-NGC-R7 | ACTATCGGTCTCACCGATGCACCAGCCGGGAATCGAACCC |  |  |
| xCas9-NGC-F8 | ACTATCGGTCTCATCGGCCACCGGCTTCGGCATGTTTCAGAGCTATGCTGGAAACAG |  | for NGC-6 |
| xCas9-NGC-R8 | ATTTGTAAGCTTAAAAAAAAAAAAGCACCGACTCGGTGCCACTTTTTC |  |  |
| xCas9-GAG-nF1 | ACTACAGGTACCGAAGCAACTTAAAGTTATCAGG | Construction of xCas9-GAG without tRNA |  |
| xCas9-GAG-nR1 | ACTACAGGTCTCGGACGGCCACGGATCATCTGCACAACTC |  |  |
| xCas9-GAG-nF2 | ACTACAGGTCTCCCGTCATTCCTGGAGAAGGTGGTTTCAGAGCTATGCTGGAAACAG |  | for GAG-1 |
| xCas9-GAG-nR2 | ACTACAGGTCTCGCTGAGCCTCAGCGCAGCAGCTTA |  |  |
| xCas9-GAG-nF3 | ACTACAGGTCTCCTCAGCCAACCACCTCTTCCGCCACGTTTCAGAGCTATGCTGGAAAC |  | for GAG-2 |
| xCas9-GAG-nR3 | ACTACAGGTCTCCAAGCCGGCAGCCAAGCCAGCACCCGCG |  |  |
| xCas9-GAG-nF4 | ACTATCGGTCTCAGCTTCCTCATGAACATTCAGGTTTCAGAGCTATGCTGGAAACAG |  | for GAG-3 |
| xCas9-GAG-nR4 | ATTTGTAAGCTTTAAAAAAAAAAAAGCACCGACTCGGTGCCACTTTTTC |  |  |

**Supplementary Table S2 |** Primers used in for identification of transgenic T0 plants and target mutation.

| Primer | Sequence (5’-3’) | Purpose |
| --- | --- | --- |
| xCas9-F | TCTGAAGTTCAGGATACGTGTG | Transgenic T_0_ plants detection |
| xCas9-R | GAGCTTATCGACGTCGCTATTA |  |
| GAA-1-F | GCCATTTTTCACAGGTCATCTC | Amplify the GAA-1 target site |
| GAA-1-R | GATCTGCTTCCGTTCTTCATTG |  |
| GAA-2-F | TCAAGTACATCCACTCGGCG | Amplify the GAA-2 target site |
| GAA-2-R | GCCAGTCAAAGAAGCAGTGC |  |
| GAA-3-F | AACACGGTCACCAACTTCATC | Amplify the GAA-3, GAC-7 target sites |
| GAA-3-R | CCCACATGAATGATGCATATG |  |
| GAT-1-F | TTCCCTTTTTAATAGCTGCCTTC | Amplify the GAT-1 target site |
| GAT-1-R | GTGTTGTTGTAGCAGTTAGTGACAG |  |
| GAT-2-F | TTCAGGTCATCCTTCGATTTC | Amplify the GAT-2 target site |
| GAT-2-R | GATACTTCTCCTCCATGCTCTTG |  |
| GAT-3-F | GCGAAGAACTGGGAGAATGTG | Amplify the GAT-3 target site |
| GAT-3-R | ACACACATAAATTCAGGGTCCG |  |
| GAG-1-F | GCAGACGAACACAACATCCTC | Amplify the GAG-1, GAC-1 target sites |
| GAG-1-R | TAAGCACACACAAACTTCGATC |  |
| GAG-2-F | AAGACCGGCCGTAAGAACC | Amplify the GAG-2 target site |
| GAG-2-R | TACCCTGGTAGATTCATCGAGG |  |
| GAG-3-F | TACACCTACAAGCGGCCAC | Amplify the GAG-3 target site |
| GAG-3-R | AGTCCTGCCATCACCATCC |  |
| GAC-2-F | AGGTTAACTACATTTCCTCCGG | Amplify the GAC-2 target site |
| GAC-2-R | CAGAGCAAGACAGCAACAAGG |  |
| GAC-3-F | TTACGAACTTTATAACTTTGTCGG | Amplify the GAC-3 target site |
| GAC-3-R | ATGGAGGCGATGAGGAAGAC |  |
| GAC-4-F | GTAAGAACCACCAGCGACAC | Amplify the GAC-4, GAC-6 target sites |
| GAC-4-R | AAGCAACAGGTCAGCCTTATCC |  |
| GAC-5-F | CCCAAGAAACTGCTCCTTAAGTCC | Amplify the GAC-5, NGC-6 target sites |
| GAC-5-R | GAGCGATCGAAGTTTGTGTG |  |
| GAC-8-F | GAGTTCACTTTTAAGTGTTTATGCGG | Amplify the GAC-8 target site |
| GAC-8-R | AGTCCGGGATAGTCTTGTAAACAG |  |
| NGG-1-F | GCTGAATGCGAAATGCGATC | Amplify the NGG-1 target site |
| NGG-1-R | GCTGGGATCAAACACAAGC |  |
| NGG-2-F | TCAAGCTCCTCAGGCACCTC | Amplify the NGG-2 target site |
| NGG-2-R | GCCAGTCAAAGAAGCAGTGC |  |
| NGA-1-F | CATCTATTGAAAATCCATGGCG | Amplify the NGA-1, NGA-2 target sites |
| NGA-1-R | CATAGCAAGCAGAGGCTGATC |  |
| NGT-1-F | GAGACCTCAGGCCAAGTAATTTG | Amplify the NGT-1 target site |
| NGT-1-R | CAAGTATGGATGGCACAGAGC |  |
| NGT-2-F | TCAAGCTCCTCAGGCACCTC | Amplify the NGT-2 target site |
| NGT-2-R | CAGCAAGCACGAGTTGGG |  |
| NGC-1-F | CCTCATCGCCTTGCTGTC | Amplify the NGC-1， NGC-4 target sites |
| NGC-1-R | CAACCAGTCAACCACTACGCTG |  |
| NGC-2-F | CATGTGGTTGGCTCATTGG | Amplify the NGC-2, NGC-5 target sites |
| NGC-2-R | GGATGCAGCCTACCGACC |  |
| NGC-3-F | CTAATTCGTGTATTGATGCTACCTG | Amplify the NGC-3 target site |
| NGC-3-R | AGAGGTTCAGCTCAGGGTAATCC |  |

**Supplementary Table S3 |** The Locus ID of rice genes.

| **Target gene** | **Locus ID** |
| --- | --- |
| *OsMPK2* | LOC_Os08g06060 |
| *OsMPK5* | LOC_Os03g17700 |
| *OsNRT1.1B* | LOC_Os10g40600 |
| *OsWaxy* | LOC_Os06g04200 |
| *OsALS* | LOC_Os02g30630 |
| *OsGRF4* | LOC_Os02g47280 |

**Supplementary Table S4 |** Mutation frequencies at GAG PAM sites by CRISPR-xCas9 system without tRNA.

| **PAM sequence** | **Target site** | **Target gene** | **Tested T_0_ plants** | **Edited T_0_ plants** | **Mutation frequency (%)** |
| --- | --- | --- | --- | --- | --- |
| GAG | GAG-1 | *OsWaxy* | 20 | 7 | 35 |
|  | GAG-2 | *OsALS* | 20 | 0 | 0 |
|  | GAG-3 | *OsALS* | 18 | 7 | 38.9 |
